# A Case of Peripheral Neuropathy From Contact With Military Radar Equipment

**Published:** 2009-12-11

**Authors:** Mayer Tenenhaus, Bruce Potenza, Andrew Li

**Affiliations:** ^a^ Division of Plastic and Reconstructive Surgery, University of California, San Diego (UCSD) Medical Center; ^b^ Division of Burn Surgery, UCSD Medical Center, San Diego, California; ^c^ UCSD School of Medicine, San Diego, California.

## Abstract

Complex early and late neuropathies resulting from direct and conductive electrical patterns of injury are disabling and extremely challenging to manage. We report a patient who suffered acute onset of right-hand weakness and decreased sensation following a radar equipment–related electrical injury. He received a carpal and Guyon tunnel release, regaining sensation and strength to his right hand by the first postoperative day.

Radar-induced injuries are often associated with burns that involve excess irradiation of tissues with high-energy electromagnetic radiation. Electricity, induced from electromagnetic waves, can cause extensive damage to many tissue types, depending on the nature of the energy (high or low voltage, alternating current vs direct current) as well as the conductive nature of the tissue through which the electricity is flowing. In this article, we report on a patient who suffered an electrical burn following contact with a high-energy military radar antenna and discuss possible mechanisms of injury that might have occurred.

## CASE REPORT

A 23-year-old, previously healthy man, on active naval duty, came in close contact with a high-energy radar antenna transformer and received an electrical injury that left burn marks on his right hand and foot. The patient was working with his hands positioned at approximately 4 to 6 inches from the transformer and saw a blue arc of electricity going from the transformer to his right hand. He was instantaneously thrown back and endorsed subsequent pain in his right upper and lower extremities. The transformer had been carrying an electrical potential of 18,000 V. Its operating frequency, deemed classified information, cannot be reported. However, typical frequencies of operation aboard naval sea craft on radar equipment range from 400 to 10,000 MHz.^1^ Later, a discharge burn was found on the transformer, which was evidence that an electrical discharge had indeed occurred between the patient and the transformer.

On admission and physical examination, some “white ash” was noted on the skin overlying the metacarpophalangeal joints and the presumed electrical exit site presented as a 1-cm full-thickness eschar on his foot. The patient had limited voluntary motion of his right arm, shoulder, and elbow, as well as a tingling sensation over his right wrist. On provocative testing, the patient was noted to have decreased active flexion of all of his fingers. The extremity was soft, well perfused with brisk capillary refill to all digits. Compartment pressures were not elevated. Noteworthy laboratory values consisted of a serum creatine kinase level of 1100 U/L (normal 0–175 U/L). Neck, chest, and extremity radiographs did not reveal any evidence of fracture or dislocation.

The patient was admitted to the burn intensive care unit, strict cardiac monitoring, and judicious hydration instituted. The affected extremities were carefully elevated and serially evaluated for compartment syndrome or progression. There was neither worsening nor improvement in the patient's neurologic status. There was no evidence of myoglobinuria. We discussed with the patient operative exploration and decompression, and on the following day the patient was taken to the operating room for exploration of the right upper extremity with median and ulnar nerve decompression.

Intraoperatively, all neurovascular, tendinous, and muscular anatomy were found to be without any signs of overt damage. A combined release of carpal tunnel and Guyon tunnel was performed through the same incision, and both nerves followed to the level of the proximal forearm and elbow. Prior to skin closure, the digits were all gently ranged and all involved tendons were noted to have normal excursion. Postoperatively, the patient did very well, and in the following day, the sensation and range of motion in his right hand dramatically improved. He was discharged on the fourth postoperative day. Subsequent follow-up found the patient to be healthy, active, and without neurologic or visual impairment.

## DISCUSSION

### Epidemiology

In the United States as of 1999, electrocution was found to be the fifth leading cause of death in the workplace.^2^ Electrical injuries occur most commonly in electrical workers,^3^ and of these, radiofrequency burns and electrocution constitute an uncommon proportion of these injuries.^3^,^4^ Because of the pervasive nature of handling of electrical equipment on naval ships,^5^ current naval safety manuals stress the importance of using electrical safety shoes, rubber safety gloves, and safety shorting probes for the safe discharge of capacitors in electrical equipment, among other preventative measures.^5^ Deck-insulating material is installed on naval ships around areas where electricity-related work is done,^5^ in an effort to protect not only the technician but also other uninsulated shipmates who happen to be in the work area.

Unlike conventional work environments, performing tasks aboard naval sea craft presents unique challenges to those aboard. Parrish et al^6^ suggest that the unfamiliar work environment aboard naval ships likely leads to high rates of injuries observed at the start of each aircraft carrier mission, especially in younger, less experienced personnel who are often given the most physically difficult jobs to perform. Deployment presents even more challenges with respect to safety, consisting of irregular work hours and near daily confinement of personnel to the ship upward of 6 months at a time.^7^ Not only does this make it more difficult for injured crew members to be delivered off the ship and replaced with healthy personnel,^7^ the monotonous environment itself may lead personnel to seek leisurely activities in areas where play and recreation were not designed for or intended. It has also been suggested that sailors may resort to higher risk-taking behavior to relieve boredom,^6^ a likely consequence of the monotony of ship life.

Review of epidemiological data obtained from the Naval Safety Center revealed that since 2000, there were a total of 990 reported electrical injuries aboard naval ships, constituting approximately 69% of total injuries reported. Interestingly, in that period only 2 radar-related injuries were reported, both of which involved exposure of the sailors to undue amounts of radiation.

### Basic radar science

Radar transmitters propagate and transmit high-power RF waves through the creation of a high-voltage and high-frequency alternating electrical current.^8^ At any given time, radar antennae can carry high voltage at a high frequency. Antenna receivers function nearly reciprocally compared with their transmitter counterparts in that they are bathed in an external electromagnetic field, the influence of which generates local charge separation and hence currents in the (antenna) wire.^9^ The eventual effects of these local charge separations and current flow are to generate voltage fluctuations proportional to any modulation imposed on the incident wave,^9^ and these fluctuations can then be processed into detected signals. The received voltages are typically extremely small, around 0.1–10 µV and, therefore, pose no health threat to the user. It is the transmitter power that poses potential health threats.

### Tissue injury through electrical current flow in tissue

Hocking et al^4^ discuss a 53-year-old man who was handling a TV transmitter and subsequently experienced an electrocuting event at 196 MHz of alternating current. The patient witnessed 2 blue flashes, although he did not experience any sensation of shock or burn and was able to walk away from the event. In the days and months following the event, the patient developed a variety of curious symptoms, beginning with a 3- to 4-cm bilateral rash on his lower rib cage on the second day after the incident to swelling, aching, and pain in his hands, wrists, and elbows by the end of the week, to the sensation of pins and needles in either hand when holding objects such as the telephone by 2 months, and to shooting pains in his forearms and recurrence of metacarpophalangeal (MCP) joint swelling by the end of 8 months.^4^

The authors speculate that these neurocutaneous symptoms may have been mediated through vascular injury from electrical current. Unlike lower frequency currents, such as the standard household 50 Hz alternating currents, higher-frequency currents mainly flow on the surface of conductors rather than within them, that is, the skin effect.^4^ This, in turn, may have led to current flowing in superficial veins and capillaries, leading to vascular endothelial dysfunction, resulting in either vasoconstriction or vasodilation.^4^ The authors speculate that the patient's sensation of “pins and needles” improved after ultrasound therapy possibly because of ultrasound's ability to reverse vasoconstriction. Moreover, the authors state that the improvement of the patient's symptoms with phenoxybenzamine and nitrates but not clonidine supported a vascular mechanism for the development of the patient's symptoms of “pins and needles.”

The effect of electrical current flow on nerve function is also interesting. It has been observed that about 30% of patients who suffer from electrical injuries also suffer some form of peripheral nerve injury.^10^ There exist quite a few theories explaining the pathophysiology behind electrical injury and the subsequent development of neuropathies. These include thermal damage, sympathetic stimulation, vascular damage, histological and electrophysiological changes, and direct mechanical trauma.^11^ Smith et al^11^ organized these mechanisms into temporal categories, which are “a delayed-reversible neuropathy: caused by thermal injury to the perineural tissues, resulting in a progressive perineural fibrosis and a correctable compressive neuropathy,” “an immediate-transient neuropathy: caused by the nerve's conduction of electricity with resultant reversible histological and electrophysiological changes…,” and an “immediate-irreversible neuropathy: caused by direct thermal damage within the nerve, with resultant necrosis.”

Fan et al^10^ used peripheral nerves in rats to investigate a nonthermal mechanism for peripheral nerve damage, characterized by very short durations (10 milliseconds) of high-voltage currents, allowing for minimal heat generation. Their use of insulated needle electrodes essentially eliminated perineural tissue damage, thereby generating a purely nerve-related model. They concluded that these short pulses of high-voltage currents caused “axolemma detachment and cell invagination,”^10^ a finding that supports the theory that such short durations of electrical current through cells result in nonthermal mechanism of cell injury that is “relatively restricted to the cell membrane,”^12^ and a process called electroporation, one that tends to favor cells of larger volume that provide more plasma membrane surface area.^13^

The distinction between low-frequency and high-frequency alternating current injury is an important one. Lee et al^3^ explain that at low-frequency current, tissue conductivity becomes the determining factor of electric current distribution. Favored tissues include major arteries and nerves when studied in a canine model.^14^ Microscopically, “low frequency current distribution in cells is determined by the density, orientation, shape, and size of cells.”^4^ Notably, cell membranes are what make tissues less conductive, and so, larger cells offer proportionately less cell membrane to protect the cytoplasmic contents of cells from conducting a low-frequency current.^4^ In contrast, the cell membrane is no longer a protective factor against current conduction when dealing with higher-frequency electrical injury in the ranges of radiofrequency and microwave frequency.^4^

### Tissue injury through thermal effects

Under the influence of radiofrequency, charges can undergo movement within tissues themselves. Electromagnetic waves like radiofrequency can move charged molecules encountered in the propagating electromagnetic field. Increased molecular motion leads to the local production of heat through frictive forces^15^ that is associated with increasing temperature. Moreover, the resultant movement of charged particles within the local irradiated tissue creates “eddy”^15^ currents, which then flow through the skin's inherent resistance and are dissipated as heat.^15^ At a certain point, whether dictated by time of exposure or the amount of heat applied to the skin, tissue damage ensues.

Thermal damage on nerve fibers themselves has been studied by Xu et al,^16^ who found in rat sciatic nerve specimens that unmyelinated fibers displayed reversible conduction block at 47°C and whose function was immediately destroyed at 58°C. Their findings were consistent with those of Rasminsky,^17^ who observed conduction block in nerves with minor temperature elevations. Xu et al explain that nerve conduction block occurs because increased temperatures lead to a decrease in sodium influx into the axon and an increase of potassium outflow from the axon. This ultimately leads to a faster repolarization and a decreased action potential propagation of the axon.^18^–^20^ The reversibility observed in the conduction block was likely secondary to the observed increased unmyelinated nerve fiber blood flow, which likely helped dissipate the heat that was causing the ion channel dysfunction.^16^

It is interesting to note that myelinated nerve fibers, when warmed to 47°C, underwent delayed conduction failure only after nerve temperature had settled back down to normal.^16^ Xu et al further noted that the nerve blood flow recorded in these myelinated fibers decreased during the warming process and that myelinated nerve fiber function was preserved during this time. Subsequent morphologic study of the vasa nervorum of these fibers revealed thromboses, which likely explained the delayed nerve dysfunction. The authors suggest that nerve blood vessels are more sensitive to thermal damage than are the nerve fibers themselves, and that myelinated nerve fibers are likely more susceptible to ischemia than are their unmyelinated nerve fiber counterparts.

### Tissue injury through perineural injury

Thermal damage to perineural structures has been observed in histologic investigations by DeBono^13^ of upper extremity of a patient who suffered a high-voltage (100,000 V) upper electrical injury and underwent subsequent amputation. DeBono found that more distal parts of the arm were more severely injured than the proximal parts. Histology taken from the wrist “showed a significant degree of neural damage with coagulation of the perineurium and the surrounding tissues being very evident.”^13^ From their own histological investigation of the nerve specimens taken from low-voltage injured nerve specimens, Smith et al^11^ suggest “that the electrical injury causes maximal heat production at areas of minimal limb cross-sectional area. In these areas, the peripheral nerve is in close proximity to bone and fibrous tissue. This results in perineurial fibrosis and symptoms of a compressive peripheral neuropathy.”^11^

### Tissue injury through contact with conductive objects

An individual can develop an electrical burn by contacting with a conductive object in which a voltage is induced by external radiofrequency waves.^5^ Interestingly, radiofrequency-induced voltage has been observed in different contexts. Nakamura et al^21^ reported that induced voltage was seen in conductive loops when they were placed in a whole-body magnetic resonance (MR) system and were irradiated with radiofrequency. They concluded that “simple loops of conductive material may result in the induction of a large and potentially hazardous voltage in the imaging system.”^21^ It is very plausible that conductive objects that happened to be near a source of high-energy radiofrequency may develop induced voltages and currents. The subsequent contact of an individual with this charged conductor may then trigger an electrical burn (Table 1).

### Treatment

Similar to our particular case study, Smith et al^11^ documented 3 patients who suffered low-voltage (<1000 V) alternating-current electrical injuries to their hands, and, as a result, developed immediate symptoms and signs resembling peripheral nerve compression without clinically significant cutaneous burns. The symptoms persisted and worsened in some respects, particularly with pain. Smith et al eventually treated these patients with nerve decompression surgery 7 months, 9 months, and 3 years after each of the patients' electrical injury event, respectively. It was found that these procedures effectively relieved some of the symptoms of neuropathy.

## CONCLUSION

Despite the significant number of electrical injuries that occur on naval vessels, radar-induced electrical injuries thankfully have been relatively infrequent. The potential for significant injury continues to pose a challenge in this high-tech, high-energy, and dynamic environment. It is difficult to specifically delineate the exact etiology of our patient's neuropathy. The dramatic response to early surgical decompression suggests a limited and reversible neuropathy. Related mechanisms of injury as described above likely contributed as well, such as thermal damage and electroporation. Further education regarding electrical injuries from radar systems should be encouraged especially among personnel aboard naval sea craft, who will inevitably encounter these potentially hazardous pieces of equipment. Further study and experience treating these injuries will hopefully lead to better prevention and treatment regimens.

## Acknowledgments

We thank Dr Gabriel M. Rebeiz, professor, Department of Electrical Engineering and Computer Science, School of Engineering, the University of California, San Diego, for his knowledge and time in editing the portions of the article regarding the physics of radar.

## Figures and Tables

**Table 1:** Mechanisms of electrical injury leading to peripheral neurological dysfunction

Electrical current flow through tissue							
Vascular injury	Neural injury	Perineural injury	Thermal injury	Induced voltage in conductive materials	Radiofrequency-induced current within tissues
High-frequency current flows along the skin (skin effect) leading to endothelial damage in superficial veins and capillaries, leading to “pins and needles” sensation	*Electroporation*: axolemma detachment and cell invagination as a result of electrical current effects on neuronal cell membrane	Electricity flows through tissue compartments and causes perineural tissue fibrosis, leading to compressive neuropathies	With increased temperature, unmyelinated neurons can undergo reversible conduction block through decreased sodium influx and increased potassium outflow, decreasing production of action potentials	External radiofrequency waves induce a voltage potential in nearby conductive objects. A patient contacts these objects and subsequently suffers an electrical burn. See “Electrical Current Flow Through Tissue”	Radiofrequency waves travel through tissue and move charged particles in the tissue, creating an internal current, which generates heat from frictive forces and subsequent thermal injury. See “Thermal injury”
			Myelinated neurons experience delayed conduction block likely through thrombosis of vasa nervorum and their greater susceptibility to ischemia than unmyelinated neurons		
